# A sex‐ and gender‐informed future for Parkinson's disease care

**DOI:** 10.1002/ctm2.70382

**Published:** 2025-06-23

**Authors:** Mariapaola Barbato, Beatriz Guzman, Santosh Dixit, Antonella Santuccione Chadha, Roberta Marongiu

**Affiliations:** ^1^ Women's Brain Foundation Basel Switzerland; ^2^ Innovation Labs, Persistent Systems Pune India; ^3^ Department of Neurosurgery Weill Cornell Medicine New York New York USA; ^4^ Department of Genetic Medicine Weill Cornell Medicine New York New York USA; ^5^ Feil Family Brain and Mind Institute, Weill Cornell Medicine New York New York USA

## 1 DEEPENING OUR UNDERSTANDING

**FIGURE 1:**
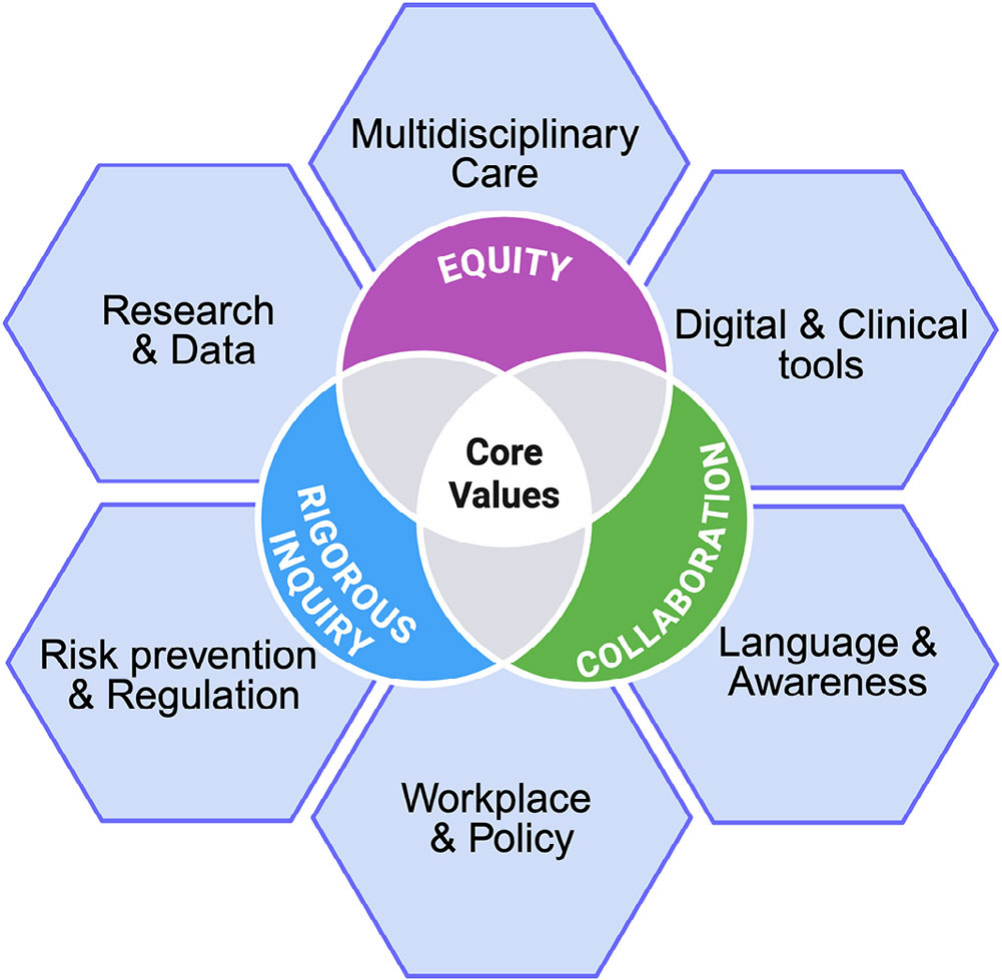
Strategic directions. Framework for sex‐ and gender‐informed Parkinson's disease research & care interweaves six domains—Language & Awareness, Digital & Clinical Tools, Multidisciplinary Care, Workplace & Policy, Research & Data, and Risk Prevention & Regulation—underpinned by the core values of equity, collaboration and rigorous inquiry.

Biological sex and its accompanying hormonal milieu profoundly shape Parkinson's disease (PD) risk and trajectory.^1^, ^2^ Higher lifetime estrogen exposure appears neuroprotective, while menstrual‐cycle fluctuations and the transition through menopause can significantly modulate PD motor and non‐motor symptoms. Conversely, men frequently experience earlier motor onset and more rapid cognitive decline, indicating a distinct clinical course.^1^, ^3^ Beyond biology, sociocultural gender norms influence who seeks care, how symptoms are reported, and which resources are accessible. Women with PD often face the longest diagnostic delays, suboptimal treatment and are less likely to access neurologist care or advanced therapies, underscoring the need to confront both biological and social determinants of health.^4^ Precision medicine mandates that we honor biological diversity and lived experience. Yet, PD females remain underrepresented in preclinical research and clinical trials, and critical hormonal influences are sidelined in research study design and clinical care.^1^, ^5^ Castro‐Aldrete et al. call for a recalibration of research priorities and clinical frameworks, embedding sex‐ and gender‐informed principles from discovery through policy to achieve equitable, personalized PD care (Figure 1).

## 2 BRIDGING THE CLINICAL GAP

Although awareness of sex and gender influences in PD is growing,^6^ clinical practice remains compartmentalized and continues to lack a sex‐ and gender‐informed approach. Neurological assessments still emphasize on motor symptoms, often overlooking broader health factors, such as hormonal transitions (menstruation, pregnancy, postpartum changes, use of contraceptives and menopause) that critically shape women's symptom profiles and treatment responses.^7^ Indeed, hormonal states can alter dopaminergic neurotransmission,^3^ while pharmacokinetic studies reveal that women exhibit greater levodopa bioavailability and a higher risk of dyskinesia, reinforcing the necessity for sex‐specific dosing strategies.^8^, ^9^ Importantly, these variables are interdependent: while hormones influence symptoms and drug response, drugs may, in turn, interact with hormonal treatments; yet current care models treat them in isolation.

Structural barriers further compound the problem. Many neurologists lack the tools, time, and clinical guidance required to gather and act upon sex‐specific information. This represents both an equity issue and an opportunity to advance precision neurology. To overcome these limitations, we envision a truly integrated care model in which neurologists, gynecologists, endocrinologists, and mental‐health specialists collaborate to develop holistic treatment plans. This multidisciplinary, collaborative environment will facilitate comprehensive care plans that address both neural and systemic contributors to PD.

Such a model would be supported by clinical and digital tools, and electronic health‐record prompts designed to capture reproductive history and hormone status, symptom checklists calibrated to reflect sex‐specific and life‐stage variations, and clinical guidelines that interpret PD manifestations through the lens of perimenopause, pregnancy and even andropause in men. These resources would enable clinicians to anticipate symptom variability, tailor medication doses appropriately (based on disease stage, hormonal context, and individual health trajectories), and deepen their dialogue with patients, thereby delivering more nuanced and effective care.

## 3 BRIDGING THE RESEARCH GAP

Despite the growing acknowledgement of sex and gender differences, and funding and reporting mandates, gaps persist in research pipelines which remain skewed toward male models and participants, limiting the translatability of findings only to half of the population.

Addressing these gaps requires concerted action focusing on building infrastructure and cultivating expertise and innovative methodologies. First, establishing centralized biorepositories that pair well‐characterized biospecimens with detailed reproductive and hormonal metadata will enable retrospective and prospective analyses of estrogen, progesterone and androgen influences. Developing and disseminating standardized assays and protocols for measuring sex hormone levels in both animal models and human cohorts will also improve comparability across studies. Additionally, real‐world data registries that systematically capture menstrual cycle phases, menopausal status and hormone therapy use in people living with PD will expand our understanding of symptom variability outside clinical trial settings. Implementing adaptive clinical trial designs that stratify randomization by hormonal phase or reproductive stage can reveal phase‐specific drug responses and reduce confounding. On the other hand, incentivizing public–private consortia to co‐develop and validate sex‐specific biomarkers and digital phenotyping tools, as well as to apply predictive and generative artificial intelligence for Big Data analysis, will accelerate the translation of research from bench to bedside and tailor interventions to real‐world diversity. Furthermore, interdisciplinary training fellowships—uniting neuroscientists, endocrinologists, bioinformaticians and patient advocates—can seed a new generation of researchers fluent in sex‐ and gender‐informed methodologies. Finally, leveraging computational modelling and Artificial Intelligence approaches to integrate hormonal, genetic and environmental variables promises to predict individual trajectories, generate data‐driven actionable insights and optimize personalized interventions. By embedding these standard strategies, the research community can generate robust, generalizable data that that enhance scientific validity, broaden applicability, and guide precision therapies for both women and men.

## 4 ADDITIONAL STRATEGIC DIRECTIONS

Castro‐Aldrete et al. provide a framework for sex‐ and gender‐informed PD research and care. This model interweaves six domains—Language & Awareness, Digital & Clinical Tools, Multidisciplinary Care, Workplace & Policy, Research & Data, and Risk Prevention & Regulation—underpinned by the values of equity, collaboration and rigorous inquiry (Figure 1).

To begin, the language we use must evolve. Adopting “journey partner” in place of “caregiver” can destigmatize support roles and foster more egalitarian relationships. Clinician education should emphasize sex‐ and gender‐nuanced presentations of PD, equipping practitioners to recognize how emotional, cognitive and non‐motor symptoms may manifest differently across sexes and life stages. Furthermore, workplace policies must evolve to support families affected by PD. Flexible scheduling and paid leave for journey partners can mitigate the disproportionate economic and emotional burdens often shouldered by women, advancing social justice in professional settings. Finally, risk prevention and regulation must address gendered environmental exposures. For instance, targeted interventions to reduce pesticide contact in female‐dominated occupations could narrow incidence gaps and inform broader public‐health policies.

## 5 CONCLUSIONS

**FIGURE 2:**
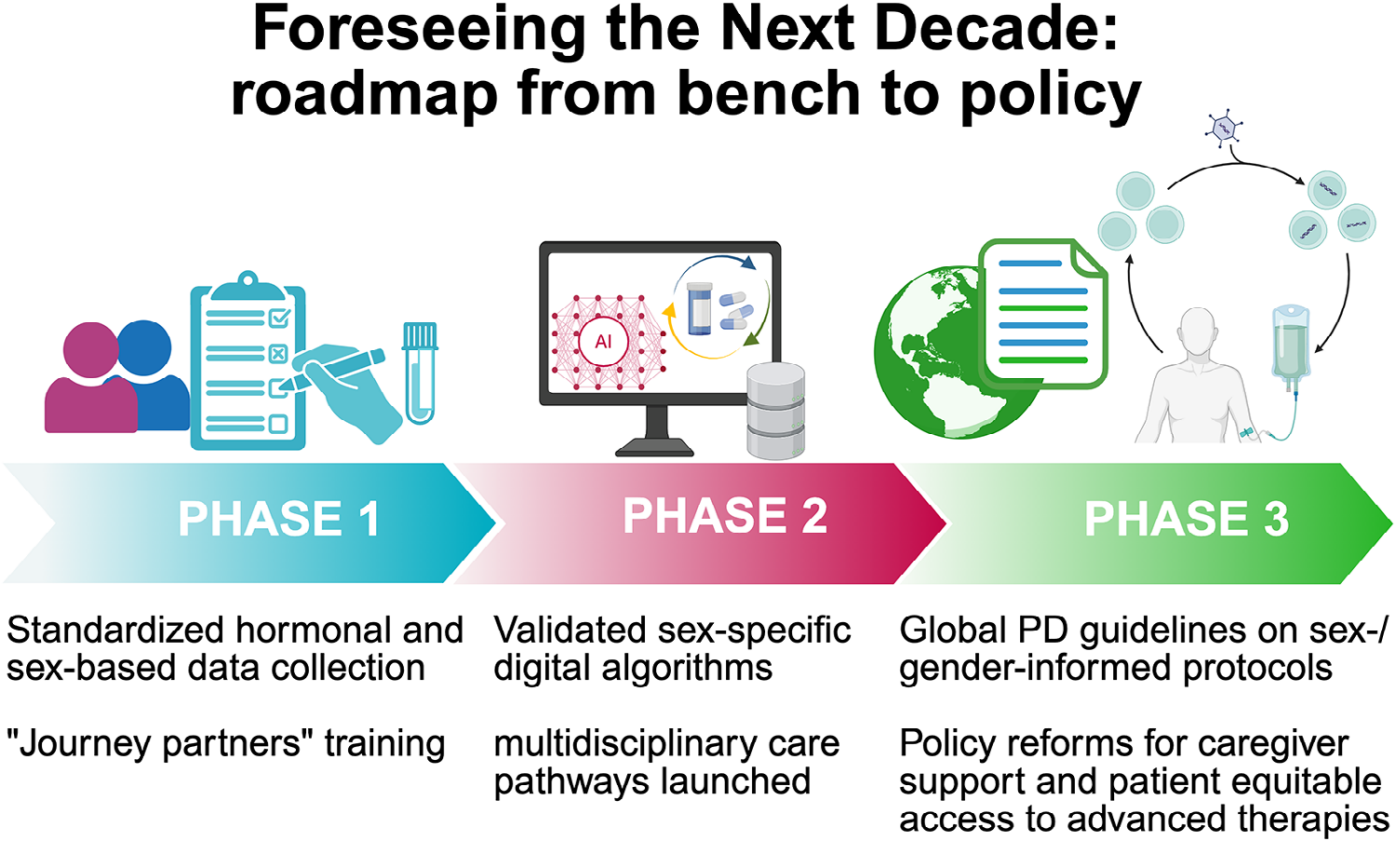
Foreseeing the next decade: from bench to policy. Ten‐year trajectory unfolds: first, hormonal and sex‐based data collection will be standardized across Parkinson's disease (PD) studies and “journey partner” training and support piloted; following, sex‐specific digital algorithms will be validated and multidisciplinay care pathways launched at academic centers; finally, global PD guidelines will enshrine sex‐ and gender‐informed protocols and policy reforms will secure caregiver support and equitable access to advanced therapies.

Centering equity, collaboration and rigorous inquiry does more than improve outcomes—it transforms the very ethos of PD care. Castro‐Aldrete et al. illuminate a future in which each person's biology and lived story guide every research question, every therapeutic trial and every policy decision (Figure 2). Embracing this vision promises more effective and personalized treatments, fewer disparities and richer lives for all who navigate the Parkinson's journey.

## AUTHOR CONTRIBUTIONS

MB: writing original draft, reviewing and editing, conceptualization; BG, SD, and ASC: reviewing and editing the draft; RM: writing original draft, reviewing and editing, conceptualization, visualization.

## ETHICS STATEMENT

No human subjectes were involved in the preparation of the manuscript. The authors declare no potential conflicts of interest.
